# Antiviral Activity of Liposomes Containing Natural Compounds Against CHIKV

**DOI:** 10.3390/pharmaceutics17091229

**Published:** 2025-09-22

**Authors:** Marília Freitas Calmon, Luiza Araújo Gusmão, Thalles Fernando Rocha Ruiz, Guilherme Rodrigues Fernandes Campos, Gabriela Miranda Ayusso, Tamara Carvalho, Isabella do Vale Francisco Bortolato, Pâmela Joyce Previdelli Conceição, Sebastião Roberto Taboga, Ana Carolina Gomes Jardim, Andres Merits, Paula Rahal, Antonio Claudio Tedesco

**Affiliations:** 1 Institute of Biosciences, Letters and Exact Sciences, São Paulo State University (UNESP), São José do Rio Preto 15054-000, SP, Brazil; thalles.ruiz@unesp.br (T.F.R.R.); gabriela.ayusso@unesp.br (G.M.A.); tamara.carvalho@unesp.br (T.C.); isabella.bortolato@unesp.br (I.d.V.F.B.); pamela.joyce@unesp.br (P.J.P.C.); sebastiao.taboga@unesp.br (S.R.T.); jardim@ufu.br (A.C.G.J.); p.rahal@unesp.br (P.R.); 2 Center of Nanotechnology and Tissue Engineering, Photobiology and Photomedicine Research Group, Faculty of Philosophy, Sciences and Letters of Ribeirão Preto, University of São Paulo, Ribeirão Preto 14040-901, SP, Brazil; luizaaraujo@usp.br; 3 Laboratório de Pesquisas em Virologia (LPV), Faculdade de Medicina de São José do Rio Preto (FAMERP), São José do Rio Preto 15054-000, SP, Brazil; guilherme.campos@unesp.br; 4 Laboratory of Antiviral Research, Institute of Biomedical Science, ICBIM/UFU, Uberlândia 38405-302, MG, Brazil; 5 Institute of Bioengineering, University of Tartu, 50090 Tartu, Estonia; andres.merits@ut.ee

**Keywords:** CHIKV, liposomes, berberine, emodin, antiviral, cell lines

## Abstract

**Background/Objectives:** Chikungunya virus (CHIKV), a mosquito-borne single-stranded RNA virus belonging to the genus *Alphavirus* (family *Togaviridae*), causes large-scale outbreaks. However, no specific treatment for CHIKV infections is currently available. Berberine and emodin are plant-derived compounds with anti-CHIKV activities. This study aimed to evaluate the antiviral efficacy of liposomes containing berberine (LB) or emodin (LE) against CHIKV in vitro, since nanocarriers incorporating zwitterionic polymers are known to enhance the biostability, biocompatibility, and therapeutic efficacy of drug candidates. **Methods:** Liposomes were synthesized and characterized, and cell viability was assessed to determine appropriate concentrations for subsequent assays. Confocal microscopy, antiviral assays, and western blotting were performed in BHK-21 and Huh7 cells. **Results:** In BHK-21 and Huh7 cells, LB and LE were well tolerated at concentrations of 5 and 10 µM, respectively. In both cell types, liposomes were internalized; LE was predominantly localized in the cytoplasm, whereas LB was also detected in the nucleus. EGCG, used as a standard drug against CHIKV in antiviral assays, exhibited virucidal activity and inhibited RNA replication and multiple stages of the CHIKV replication cycle in BHK-21 and Huh7 cells. Both the nanoformulations and EGCG consistently suppressed the expression of CHIKV replicase and virion proteins. **Conclusions:** These findings highlight the potential of berberine- and emodin-loaded liposomes as antiviral agents against CHIKV infection.

## 1. Introduction

Chikungunya virus (CHIKV) is a mosquito-borne *Alphavirus* (family *Togaviridae*). It has a positive-sense single-stranded RNA (+ssRNA) genome of approximately 12 kb, which can function directly as mRNA upon infection, featuring a 5′ cap and a poly A tail, and contains two open reading frames (ORFs) that encode non-structural and structural proteins [1]. The nonstructural protein nsP1 is involved in the formation of the cap structure, whereas nsP2 functions as an RNA helicase and a protease. nsP3 mediates interactions with host proteins, and nsP4 exhibits RNA-dependent RNA polymerase activity. Structural proteins, including capsids, 6K/TF, and envelope glycoproteins, are required for viral particle formation [2].

The virus was first isolated from the serum of an infected patient in Tanzania in 1952 [3]. CHIKV has three genotypes: West African (WA), East/Central/South African (ECSA), and Asian genotypes. The ECSA genotype is divided into sublineages, including the Indian Ocean Lineage (IOL). CHIKV is globally dispersed, with the ECSA and Asian genotypes being the most prevalent [4], and it continues to cause significant outbreaks. The virus has adapted to the urban transmission cycle, posing a relevant risk to tropical and temperate regions. Two vaccines have recently been approved to prevent CHIKV infection [5].

CHIKV infects various cell types, including dendritic cells, macrophages, synovial fibroblasts, endothelial cells, and myocytes. In humans, it infects osteoblasts, contributing to joint pathology and erosive disease in individuals with chronic arthritis [6]. It is estimated that approximately 30–40% of infected individuals experience long-term sequelae, with severe pain occurring in approximately 37% of those with persistent arthralgia [7]. Although CHIKV is now endemic in many regions, no specific treatment is currently available, and treatment for CHIKV-infected patients is mainly limited to anti-inflammatory drugs for symptom relief. Therefore, developing new strategies to treat CHIKV infections is crucial.

**Figure 1:**
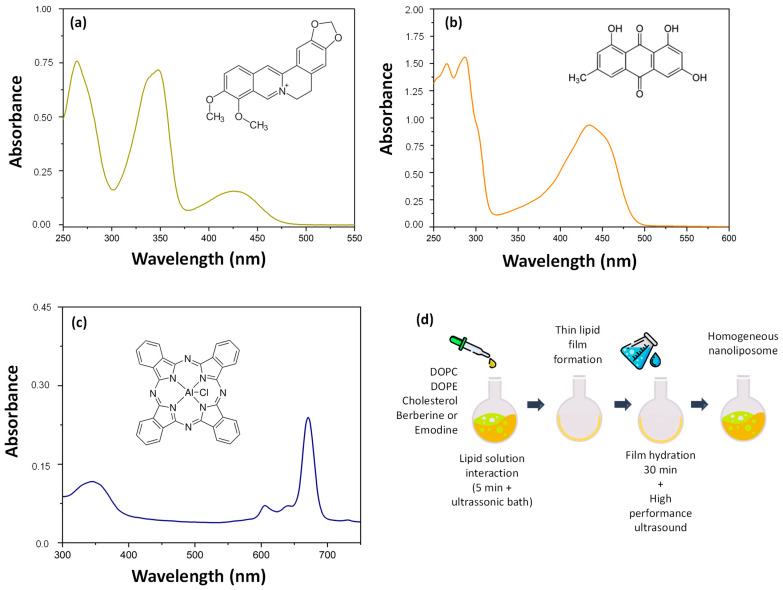
Chemical structures and UV–vis absorption spectra of (**a**) berberine, (**b**) emodin, and (**c**) chloroaluminum phthalocyanine. (**d**) Schematic representation of liposome manufacturing by the thin film formation method.

Berberine is a natural isoquinoline alkaloid (Figure 1a) found in several medicinal plants, including *Berberis vulgaris*, *Coptis chinensis*, *Hydrastis canadensis*, *Coptidis rhizoma*, *Xanthorhiza simplicissima*, *Phellodendron amurense*, and *Chelidonium majus* [8,9]. It exhibits a range of unique biochemical and pharmacological activities, including antiviral properties [10,11,12,13]. Berberine exhibits virucidal activity against dengue and Zika viruses, both RNA viruses from the family Flaviviridae, and chikungunya virus, an RNA virus from the family *Togaviridae* [11]. In addition, BBR inhibits the replication of the influenza virus, an RNA virus from the Orthomyxoviridae family, in human pulmonary adenocarcinoma cells and mouse lungs by suppressing infection [14]. BBR also impedes the attachment and entry/fusion steps of HCV, an RNA virus from the family Flaviviridae, by potentially interacting with the HCV E2 glycoprotein in Huh7.5 cells [10].

Emodin is a naturally occurring anthraquinone derivative (Figure 1b) and an active component of plants used in traditional Chinese herbal medicine [15]. It possesses a broad spectrum of pharmacological effects, including antiviral activity [16,17]. Emodin exhibited antiviral effects against HSV-1 and HSV-2 in VERO cells [18]. Emodin effectively inhibited EV71 replication in MRC5 cells, protecting them from -induced cytopathic effects by inhibiting viral maturation and diminishing cell cycle arrest in the S phase [19]. In addition, emodin reduced the infectivity of ZIKV by approximately 83.3% in Vero E6 cells [20].

However, both compounds have certain limitations, such as toxicity, poor aqueous solubility, low absorption, and limited bioavailability [21,22,23,24,25,26,27]. Therefore, it is essential to employ advanced delivery systems, such as nanoparticles, to overcome these limitations and enhance the efficacy of these compounds [24]. Nanoparticles and liposomes containing berberine and emodin improved and enhanced their pharmacokinetic parameters, promoting the use of these nanobased drug delivery systems in various treatments [28,29,30,31,32,33,34].

Owing to their charge neutrality and superhydrophilic nature, zwitterionic polymers have recently been established as materials for constructing stealth nanoparticles [35]. The self-assembly of zwitterionic polymers and copolymers into nanoaggregates, such as micelles, vesicles, liposomes, nanogels, lamellae, cylindrical micelles, and bilayers, has become a popular approach for therapeutic drug delivery. Nanocarriers incorporating zwitterionic polymers exhibit exceptional resistance to nonspecific protein adsorption. They can overcome physiological barriers, including rapid blood clearance, nonspecific tissue, and cell distribution, poor cellular uptake, and endosomal trapping, offering excellent biostability, biocompatibility, and improved therapeutic efficacy [36]. Therefore, this study aimed to analyze the antiviral activity of zwitterionic liposomes containing berberine and emodin against CHIKV infection in vitro and compare their efficacy with that of the free forms of these natural compounds. To date, this is the first study that evaluates the action of zwitterionic liposomes containing berberine or emodin as a nanosystem against the CHIKV replication cycle.

## 2. Materials and Methods

### 2.1. Synthesis of Liposomes

Lipids were obtained from Avanti Polar Lipids (Croda International Plc., Princeton, NJ, USA). Berberine chloride and emodin were purchased from Sigma-Aldrich (St. Louis, MO, USA) and dissolved in 100% acetonitrile (Sigma-Aldrich, St. Louis, MO, USA) and used to prepare berberine-containing liposomes (LB) and emodin-containing liposomes (LE) using the lipid film hydration method (Figure 1d).

In the first step, DOPE (1,2-dioleoyl-sn-glycero-3-phosphoethanolamine), DOPC (1,2-dioleoyl-sn-glycero-3-phosphocholine), and cholesterol were dissolved in 10 mL of a 4:1 ethanol-to-chloroform solution and placed in an ultrasonic bath (Ecosonics, Indaiatuba, Brazil) for 15 min. Subsequently, berberine (2.68 mM) or emodin (3.70 mM) was added to the lipid solution and sonicated for 5 min. The solution was transferred to a round-bottom flask and coupled to a rotary evaporator (Büchi Labortechnik AG, Flawil, Switzerland) operated in a water bath at 50 °C to gradually evaporate the solvent and form a thin, homogeneous lipid film on the inner surface of the flask.

In the second step, the film was placed in a desiccator (Qualividros, Passos, Brazil) for 1 h and hydrated with 10 mL of filtered ultrapure water. The flask was kept in a water bath at 50 °C and rotated at 120 rpm in a rotary evaporator (without vacuum) for 30 min. The resulting liposomal solution was transferred to a beaker and sonicated in a high-performance ultrasonic processor under the following conditions: two cycles of 2 min at 50 W. Liposomes without any incorporated compounds (EL) were also prepared using the same approach and used as controls. All liposomes were filtered through a 0.22 μm PTFE membrane (Millipore, MA, USA) and stored at 4 °C.

### 2.2. Liposome Characterization

The samples were diluted 1:100 in ultrapure water and filtered through a 0.22 μm PTFE membrane. The hydrodynamic diameter and polydispersity index (PDI) of the liposomes were measured using dynamic light scattering (DLS) at 25 °C with a scattering angle of 173° (Zetasizer Nano ZS; Malvern PCS Instruments, Malvem, UK). The zeta potential (ζ) values were determined by electrophoretic mobility measurements performed at 25 °C.

### 2.3. Drug Content (DC) and Encapsulation Efficiency (EE)

The concentrations of berberine and emodin in the liposomes were determined using standard calibration curves in acetonitrile. The liposomal sample (150 μL) was mixed with 150 μL of DMSO, sonicated in an ultrasonic bath for 30 min, and diluted to 2 mL with acetonitrile. UV–Vis absorption spectra were recorded using a Hitachi spectrophotometer (Hitachi Ltd., Tokyo, Japan). DC was determined by measuring the absorbance at 438 nm for berberine and 435 nm for emodin.

EE was calculated using Equation (1) [37], which quantifies the proportion of the incorporated drug. To determine the amount of unencapsulated drug, the aqueous phase was separated by ultrafiltration using a 10 kDa molecular weight cut-off membrane (Microcon Ultracel YM-100, Millipore, MA, USA) at 9300× *g* for 1 h at 4 °C (Eppendorf Centrifuge 5430 R, Hamburg, Germany) [38,39].

(1) $$\left(EE\;\left(\%\right)\;=\;\left[\frac{\left(C_{T}-C_{F}\right)}{C_{Th}}\right]\times 100\right)$$

C_T_ is the total concentration of the compound, C_F_ is the concentration of the free compound, and C_Th_ is the theoretical active concentration.

### 2.4. Accelerated Stability and Shelf-Life Measurements

Accelerated stability studies were performed using a LUMiSizer^®^ analyzer (LUMiSizer, LUM GmbH, Berlin, Germany) coupled to a centrifugal system that recorded the transmission profiles at each point from the top to the bottom of the analysis container [40]. For this purpose, 400 µL of liposomes were placed in cuvettes with a maximum radial position of 130 mm and centrifuged at 175 g and 25 °C for 8 h. Transmission profiles in the near-infrared region (880 nm) were analyzed using SEPView. 5.0 software (LUM GmbH, Berlin, Germany) to evaluate the distribution rate, instability index values, and sedimentation rate.

### 2.5. Cell Lines

Baby hamster kidney fibroblast cells (BHK-21, ATCC CCL-10) and human hepatocellular carcinoma epithelial cells (Huh7) were cultured in Dulbecco’s Modified Eagle’s medium (DMEM) (Cultilab, Campinas, SP, Brazil) supplemented with 10% fetal bovine serum (Cultilab, Campinas, SP, Brazil) and 1% penicillin/streptomycin (Gibco, Grand Island, NY, USA). The cells were maintained in a humidified incubator at 37 °C and 5% CO_2_.

BHK-21 cells continuously expressing the non-cytotoxic subgenomic replicon CHIKV NCT (BHK-CHIKV NCT) [41] were cultured in DMEM supplemented with 5 µg/mL puromycin (Gibco, Grand Island, NY, USA). The cells were maintained in a humidified incubator at 37 °C and 5% CO_2_.

### 2.6. Liposome Cytotoxicity

The amount of ATP in the culture is directly proportional to the number of viable cells, allowing for the estimation of cell number. The cytotoxicity of LB, LE, and EL liposomes in BHK-21 and Huh7 cells was analyzed using the CellTiter-Glo^®^ 2.0 kit (Promega, Madison, WI, USA). For this assay, 10^4^ cells were plated per well in 96-well plates and incubated at 37 °C for 24 h. The cells were then treated with various concentrations (0.6–40 µM) of the liposomes for 16 and 48 h. After incubation, the medium containing the liposomes was aspirated, and 100 μL of CellTiter-Glo^®^ 2.0 reagent (Promega, Madison, WI, USA) was added to each well and mixed for 10 min to induce cell lysis. The plate was then incubated at room temperature for 10 min to stabilize the luminescence signal, which was measured, and the cytotoxicity of each liposome formulation was calculated.

### 2.7. Confocal Microscopy Analysis of the Uptake of the Proposed Liposomes in BHK-21 and Huh7 Cell Lines

To analyze liposome uptake in BHK-21 and Huh7 cells, liposomes containing emodin and chloroaluminum phthalocyanine (ClAlPc; Figure 1c) and liposomes containing berberine and ClAlPc were synthesized. Emodin and phthalocyanine fluorescence in the visible spectrum: Emodin emits at 535 nm upon excitation at 465 nm, whereas phthalocyanine is excited at 668 nm and emits at 700 nm. Berberine fluoresces at 555 nm, with an excitation wavelength of 421 nm.

Cellular internalization of emodin, berberine, and phthalocyanine was analyzed using confocal microscopy. For this, 10^5^ BHK-21 and Huh7 cells were plated on coverslips in 6-well plates and maintained under growth conditions for 24 h. The cells were then incubated with non-cytotoxic concentrations of liposomes for 1, 8, and 16 h at 37 °C. Following incubation, cells were washed with PBS and fixed with 4% formaldehyde in PBS for 10 min at room temperature. After fixation, the cells were washed twice with PBS, and coverslips were mounted on slides using Fluoromount^®^ mounting medium containing DAPI (Thermo Fisher Scientific, Waltham, MA, USA). Images were acquired using a confocal microscope (LEICA TCS SP8, Wetzlar, Germany). The acquired images were processed, and colocalization was determined by overlaying the fluorescence images. Cell nuclei were stained with DAPI (blue), berberine and emodin showed green autofluorescence, and ClAlPc showed red fluorescence.

### 2.8. Virus

CHIKV-NLuc is a recombinant virus derived from the CHIKV isolate LR2006OPY1, which belongs to the ECSA genotype and expresses a nanoluciferase reporter for antiviral assays. In the infectious plasmid, the cDNA of CHIKV-NLuc was controlled by the human cytomegalovirus (CMV) promoter [42].

To produce CHIKV-NLuc, 10^5^ BHK-21 cells were seeded in 24-well culture plates (TPP, Trasadingen, Switzerland) and transfected with 1 μg of CHIKV-NLuc plasmid DNA using Lipofectamine 2000 (Thermo Fisher Scientific, Waltham, MA, USA) and OPTI-MEM (a reduced serum medium) (Gibco, Thermo Fisher Scientific, Waltham, MA, USA), following established protocols [43]. The supernatant was collected 72 h post-transfection and stored at −80 °C.

Viral titers were determined using a plaque assay based on a well-established protocol [43]. BHK-21 cells (10^5^ cells/well) were seeded in 24-well plates (TPP; Trasadingen, Switzerland) and incubated for 24 h. The cells were then infected with tenfold serial dilutions of CHIKV-NLuc stock for 1 h at 37 °C. After removing the inoculum, fresh medium was supplemented with 1% penicillin/streptomycin (P/S) (Cultilab, Campinas, SP, Brazil), 1% fetal bovine serum (FBS) (Gibco, Thermo Fisher Scientific, Waltham, MA, USA), and 2% carboxymethylcellulose (CMC) (Sigma-Aldrich, St. Louis, MO, USA) was added to each well. Cells were incubated for 48 h, fixed with 10% formaldehyde (Merck, Darmstadt, Germany), and stained with 1% crystal violet (Merck, Darmstadt, Germany). Finally, viral foci were counted to determine viral titers, which were expressed in plaque-forming units per milliliter (PFU/mL).

### 2.9. Liposome Dose-Dependence Assay

To analyze the dose dependence of CHIKV infection inhibition, BHK-21 and Huh7 cells were seeded in 96-well plates at 10^4^ cells per well and incubated for 24 h. BHK-21 cells were infected with CHIKV-NLuc at an MOI of 0.1, whereas Huh7 cells were infected at an MOI of 2 in the presence of LB, LE, or EL, at concentrations ranging from 40 to 0.3 µM. After a 16-h incubation at 37 °C, antiviral activity was assessed by measuring NLuc activity. EC_50_ values were calculated using non-linear regression in GraphPad Prism 8.0.1, (Boston, MA, USA) and the selectivity index (SI) was determined using the _50_/_50_/EC_50_ ratio.

### 2.10. Investigation of the Effect of Liposomes on the Direct Inactivation of Extracellular Viral Particles (Virucidal Activity)

To assess the virucidal effect of liposomes, CHIKV-Luc at an amount corresponding to an MOI of 5 was mixed with LB, LE, or EL and incubated at 37 °C for 1 h. The inoculum was then added to BHK-21 and Huh7 cells, which had been seeded in white 96-well plates (Greiner Bio-One, Frickenhausen, Germany) at a density of 10^4^ cells/well, 24 h before the experiment. After 1 h of incubation at 37 °C, the supernatant was removed, cells were washed twice with PBS, and DMEM containing 2% FBS was added to each well. The plates were incubated for 16 h at 37 °C. Luminescence was measured as previously described. Epigallocatechin gallate (EGCG, 50 µM) was used as a positive control.

### 2.11. Time-of-Addition Assay

BHK-21 and Huh7 cells were seeded in 96-well plates at a density of 10^4^ cells/well 24 h prior to infection and treatment. Infections were conducted at a multiplicity of infection (MOI) of 0.1 for BHK-21 cells and 2 for Huh7 cells. Viral replication was evaluated by measuring nanoluciferase activity at 16 h post-infection (hpi). For the pretreatment assay, the cells were treated with liposomes for 1 or 2 h before CHIKV infection and then washed twice with PBS. CHIKV-NLuc was then added for 1 h, followed by two washes with PBS to remove any unbound virus. Subsequently, DMEM supplemented with 2% FBS was added and incubated for an additional 16 h. For the entry inhibition assay, cells were infected with CHIKV-NLuc in the presence of medium containing liposomes or free compounds for 1 h, washed twice with PBS, and incubated in fresh medium for 16 h. For post-entry assays, cells were infected with CHIKV-NLuc for 1 h, washed twice with PBS, and treated with liposomes or free compounds +2, +4, +6, and +8 h post-infection (hpi). Viral replication was assessed at 16 hpi by measuring nanoluciferase activity. EGCG (50 µM) was used as a positive control.

### 2.12. Analysis of Inhibition of Viral RNA Replication

BHK-21-CHIKV-NCT cells, which harbor CHIKV replicons expressing a puromycin resistance marker (Pac), *Renilla* luciferase, and EGFP reporters [41], were used to evaluate the effects of LB and LE on CHIKV RNA replication. Cells were seeded in 96-well plates at a density of 10^4^ cells/well. After 24 h, cells were treated with liposomes for 72 h. CHIKV RNA replication was estimated by quantifying the *Renilla* luciferase expression.

### 2.13. Analysis of CHIKV Protein Expression by Western Blotting

To evaluate the expression of CHIKV proteins, 10^6^ BHK-21 and Huh7 cells were seeded in 6-well plates. After 24 h, BHK-21 and Huh7 cells were infected with CHIKV-NLuc at MOI of 0.1 and 2, respectively, for 1 h. Subsequently, the inoculum was removed and fresh medium containing LB, LE, the respective free compounds, or EGCG (positive control) was added for 16 h. The cells were then trypsinized, collected by centrifugation, and lysed using CelLytic M buffer (Sigma-Aldrich, St. Louis, MO, USA), supplemented with a protease inhibitor cocktail (Promega, Madison, WI, USA). Protein concentrations were measured using a Pierce™ BCA Protein Assay Kit (Thermo Fisher Scientific, Waltham, MA, USA).

Equal amounts of total protein (15 µg) per sample were denatured by boiling in sodium dodecyl sulfate (SDS) loading buffer and then separated by sodium dodecyl sulfate polyacrylamide gel electrophoresis on a 10% gel. Proteins were transferred onto polyvinylidene fluoride (PVDF) membranes (Merck, Darmstadt, Germany). Membranes were blocked for 1 h at room temperature with PBS containing 5% non-fat dry milk and 0.05% Tween 20. The cells were then incubated overnight at 4 °C with rabbit polyclonal antibodies against CHIKV nsP2 or nsP3 (in-house, diluted 1:2000) and the CHIKV capsid (in-house, diluted 1:1000). Rabbit monoclonal anti-β-actin antibody (1:1000; 13E5; Cell Signaling, Danvers, MA, USA) was used as a loading control. Following overnight incubation, the membranes were washed thrice with PBS containing 0.05% Tween 20. They were then incubated at room temperature for 1 h with a horseradish peroxidase (HRP)-conjugated secondary antibody (diluted 1:2000; #31460; Thermo Scientific™, Waltham, MA, USA). Protein bands were detected using an ECL substrate and visualized using a ChemiDoc imaging system (Bio-Rad, Hercules, CA, USA). The intensities of the bands were quantified using Image Lab software v 4.1 (Bio-Rad, Hercules, CA, USA) and normalized to the β-actin levels.

### 2.14. dsRNA Intercalation Assay

A migration retardation assay adapted from Krawczyk et al. [44] was used to evaluate the ability of LB and LE to intercalate CHIKV double-stranded RNA (dsRNA). PCR was performed to amplify the 3′ untranslated region (3′UTR) of CHIKV using primers flanked by the T7 promoter sequence (forward: TAATACGACTCACTATAGGGAAGTCAGTACGCCAGTCA; reverse: TAATACGACTCACTATAGGGGGAACTCATTGCATGCTG). The 273-bp PCR product was purified using the Zymoclean™ Gel DNA Recovery Kit (Zymo Research, Irvine, California, USA) and subjected to in vitro transcription using the T7 RiboMAX™ Express Kit (Promega, Madison, WI, USA). dsRNA was obtained by annealing RNA strands of opposite polarity. A total of 15 nM dsRNA was incubated with LB or LE for 45 min and analyzed on a 1% agarose gel (TAE 1×) stained with ethidium bromide. Intercalation was confirmed by the disappearance of the fluorescent dsRNA band as the intercalating compounds competed with ethidium bromide. Doxorubicin (100 µM) was used as a positive control.

### 2.15. Statistical Analysis

All experiments were conducted in triplicate with three (cytotoxicity) or four (antiviral activity) technical replicates. CC_50_ and EC_50_ values were calculated using nonlinear regression analysis of dose-response curves (log [compound] vs. response). Statistical significance was determined using one-way ANOVA followed by Tukey’s post-hoc test using GraphPad Prism 8.0.1 (GraphPad Software, San Diego, CA, USA). Statistical significance was set at *p* < 0.05.

## 3. Results and Discussion

### 3.1. Analysis of Particle Size, Polydispersity (PDI), and Zeta Potential of LB and LE

**Figure 2:**
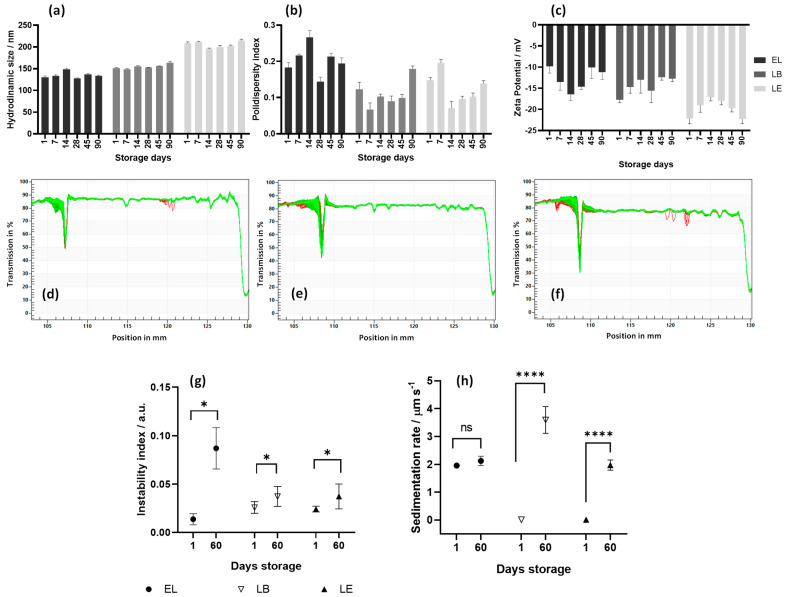
Monitoring of (**a**) hydrodynamic particle size, (**b**) polydispersity index, and (**c**) zeta potential of empty liposomes (EL) with berberine (LB) and emodin (LE) over 90 days of analysis. Accelerated stability analysis of liposomes using LUMiSizer^®^. (**d**–**f**). Transmittance profile during centrifugation. Red lines indicate the earliest profiles acquired in the LUMiSizer, whereas green lines represent the most recent ones. (**g**) Instability index and (**h**) sedimentation rate of liposomes on the first day after production and after 60 days of storage at 4 °C. EL, empty liposome; LB, liposome containing berberine; LE, liposomes containing emodin. (ns) indicates no significant difference compared to the control, whereas (*) and (****) denote a significant difference with *p* < 0.05 and *p* < 0.0001, according to Student’s *t*-test analysis.

Zeta potential, which reflects the surface charge of liposomes, and particle size are crucial parameters for liposome kinetic behavior and cellular uptake. All data was collected weekly over 90 days of storage at 4 °C. The average values of size, PDI, and zeta potential are presented in Table 1, while the weekly results obtained in technical triplicate are shown in the graphs in Figure 2a–c. As expected, the incorporation of active compounds led to a slight increase in particle size. All the samples exhibited a PDI below 0.3, indicating a consistent and uniform particle size distribution, as shown in Figure 1b.

It is important to note that the zeta potential observed in our study falls within the range of moderate colloidal stabilities [45]. Notably, this value remained constant throughout the 90 days of analysis, indicating great stability over time and no changes in the surface composition or arrangement. Literature often indicates that a zeta potential of approximately ±30 mV suggests high colloidal stability. However, it is important to recognize that liposome stability is influenced by several factors and not solely determined by the final charge of the nanoparticles. This indicated that the composition and proportion of phospholipids and steroids directly influenced the stability of these nanoformulations.

Generally, phospholipids such as DSPC, DOPE, and DOPC enhance bilayer stability, aid in preventing drug leakage, and play a key role in membrane fusion for cellular uptake. Cholesterol content significantly influences bilayer membrane fluidity and permeability. It not only enhances vesicle bilayer aggregation but also effectively fills gaps between phospholipids by facilitating hydroxyl head group interactions with the aqueous phase of the phospholipid membrane [46,47].

Although the lipids used in liposome formulations are zwitterionic, the observed negative charge is consistent with the arrangement of the functional groups on the liposomal surface. This negative zeta potential may contribute to nanoparticle stability by increasing interparticle repulsion, thereby preventing aggregation [48,49]. A moderately negative zeta potential significantly enhanced the interaction with the cell membranes, effectively promoting internalization. This process is often facilitated by proteins present in the biological environment [50,51].

Both LB and LE demonstrated encapsulation efficiencies (EEs) greater than 90%, indicating a high loading capacity, a crucial characteristic for effective drug delivery systems. This high EE is likely due to the condensing effect of cholesterol, which reduces membrane permeability and enhances the retention of the encapsulated agents [52]. A previous study reported an EE of 94.6% for calothrixin B encapsulated in liposomes composed of DOPE:DOPC:cholesterol [53], a value comparable to that observed for berberine and emodin in our study. Furthermore, liposomes composed of DOPC and DOPE have been shown to exhibit negative zeta potentials, PDI values below 0.5, and long-term (90-day) stability [53,54], which is consistent with our findings [53,54].

### 3.2. Accelerated Stability and Shelf Life of Liposomes

The accelerated stability of liposomes was assessed using the LUMiSizer^®^ by analyzing droplet sedimentation or creaming rates, as well as the instability index based on transmission profiles. The transmission profiles of liposomes one day after synthesis are shown in Figure 2a–c. Green lines represent the final profiles obtained from the analysis. The overlap of these profiles and the absence of significant changes in transmittance over time indicate the absence of phase-separation phenomena, such as flocculation, sedimentation, or creaming [55].

All samples exhibited an instability index below 0.05 (Figure 2d), suggesting excellent stability and minimal sedimentation, which would otherwise manifest as a decrease in transmittance over time [56]. Among the liposomes, the incorporation of active compounds resulted in increased stability, as evidenced by the higher sedimentation rate of EL compared to the lower rates observed for LB and LE (Figure 2e). Accelerated stability testing repeated after 60 d of storage at 4 °C revealed a slight increase in the instability indices; however, all values remained below 0.1 (Figure 2d). The sedimentation rates increased from 0.0095 to 3.59 µm/s for LB, from 0.011 to 1.97 µm/s for LE, but only minimally from 1.96 to 2.12 µm/s for EL (Figure 2e). Despite this increase, the value remained low, and the transmission profiles showed only minimal changes, confirming the sustained stability over time. These findings agree with the zeta potential and particle size data.

Based on the accelerated stability analysis data, the estimated shelf life of both the empty and loaded liposomes was approximately six months. This also suggests that storage at 4 °C is the optimal condition to prevent creaming and sedimentation while maintaining functional integrity over extended periods, which is an important consideration for potential pharmaceutical and biomedical applications. These results corroborate previous studies reporting that liposomal systems exhibit long shelf life and high stability, with variations depending on the lipid composition and nature of the encapsulated active agents [55,57,58].

### 3.3. Liposome Cytotoxicity

Liposomes containing phospholipids with short acyl chains, such as DMPC and DLPC, have been shown to destabilize cell membranes and reduce cell viability. In contrast, Bonechi et al. (2018) reported that liposomes composed of DOPE and DOPC did not exhibit cytotoxicity at any tested concentration [54]. Furthermore, long-chain phospholipids, such as DOPE and DOPC, support cell proliferation by integrating into the membrane and utilizing energy-dependent internalization pathways [59,60].

Cytotoxicity analyses of LB, LE, and EL were performed in BHK-21 and Huh7 cells. The viability of cells treated with LB and LE remained above 80% at concentrations of ≤10 µM. Cells treated with EL exhibited viability greater than 80% at concentrations ≤ 20 µM. These values were similar for both cell lines and remained consistent when the treatment duration was extended from 16 h to 48 h (Figure S1). These findings suggest that cell membrane integrity was preserved and that the antiviral effects observed in subsequent analyses were attributable solely to the encapsulated active compounds.

### 3.4. Liposomes Uptake by BHK-21 and Huh7 Cells

To investigate the cellular localization of berberine and emodin, liposomes containing berberine, ClAlPc, emodin, and ClAlPc were prepared.

To investigate the cellular localization of liposomes containing these compounds, we used particles containing ClAlPc. ClAlPc (Figure 1c) is an organic photosensitizer known for its high molar absorptivity and intense fluorescence and is widely used as a fluorescent cell marker, either in its free form or formulated into liposomes [61]. ClAlPc fluorescence was detected in the cytoplasm of BHK-21 and HuH-7.0 cells at all time points, confirming the effective cellular internalization of liposomes and corroborating previous studies that have shown that ClAlPc is predominantly localized in the cytoplasm of various cell types [59,61,62]. Based on this behavior, ClAlPc was used as a fluorescent probe to track liposomal distribution and study the release profile of berberine and emodin from the liposomal system.

**Figure 3:**
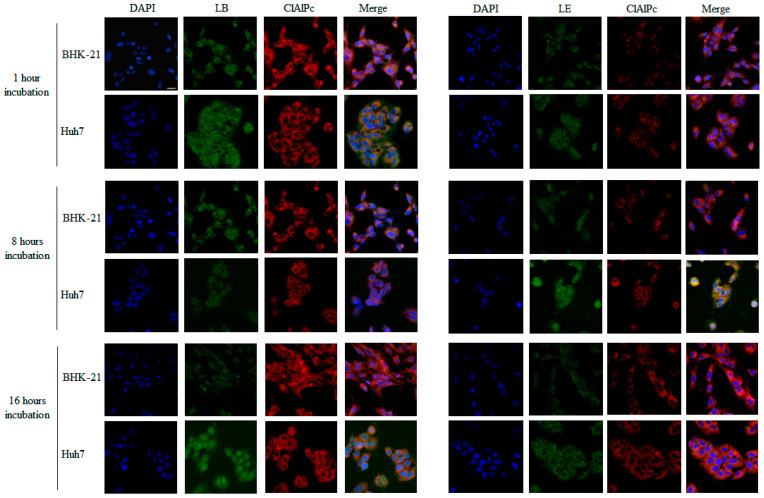
Cellular uptake and distribution of liposomes containing berberine + ClAlPc (LB), or emodin + ClAlPc (LE). Uptake by BHK-21 and Huh7 cells was analyzed using confocal microscopy at 1, 8, and 16 h after treatment. The cell nuclei were stained with DAPI (blue fluorescence), berberine and emodin showed green autofluorescence, and ClAlPc showed red fluorescence (scale bar: 20 μm).

Berberine and emodin are chromophores (Figure 1a,b). Their molecular structures feature extensive electronic conjugation, which enables them to absorb light in the visible spectrum and emit fluorescence. Consequently, both compounds could be easily visualized within the cells using confocal fluorescence microscopy. Berberine fluorescence was detected in both the cytoplasm and nucleus, with stronger signals in the cytoplasm at all evaluated time points in both BHK-21 and Huh7 cell lines treated with LB (Figure 3). Although positively charged liposomes typically show higher cellular uptake than neutral or negatively charged liposomes [62], cholesterol enrichment of negatively charged liposomes, as demonstrated in this study, can enhance cellular uptake [59]. We also observed that the berberine signal was weaker after 16 h of incubation in BHK-21 cells. The relatively weak fluorescence signal observed here can be attributed to the low concentration (5 µM) of the compound and its photobleaching properties [63].

These results are consistent with reports of berberine fluorescence in HepG2 cells after 24 h of incubation at varying concentrations [64]. Previous studies have also shown that intracellular berberine localizes to the mitochondria, nucleus, and cytoplasm in a concentration-dependent manner [65,66]. Similar findings have been reported for HepG2 cells, in which berberine fluorescence intensity increased with concentration. Another study using berberine-loaded nanoparticles (BBR-GA NPs) detected berberine fluorescence in the cytoplasm of A549 cells after 6 h of incubation [63]. Similarly, cytoplasmic localization of berberine was observed in MCF-7 cancer stem cells 4 h after treatment with 20 µM berberine-loaded liposomes [65].

Emodin cytoplasmic fluorescence was detected at all evaluated time points in both cell lines incubated with LE. However, the signal was also weak, likely due to emodin photobleaching upon exposure to UV or visible light (Figure 3) [67,68]. The fluorescence signal of emodin was weaker in BHK-21 cells after 16 h of incubation. These findings are in agreement with previous studies that demonstrated the cytoplasmic localization of emodin in HT-29 cells treated for 48 h with emodin-loaded spherical mesoporous silica nanoparticles [69], and in MCF-7 cells treated with spherical nanoparticles containing emodin for 2, 4, 12, and 24 h, where a subtle fluorescence signal was observed at 2 and 4 h [70]. Overall, confocal microscopy results confirmed that the liposomes were efficiently internalized by BHK-21 and Huh7 cells and were localized predominantly in the cytoplasm, that is, in the compartment where CHIKV replication takes place.

### 3.5. LE and LB Inhibit CHIKV Replication in a Dose-Dependent Manner

**Figure 4:**
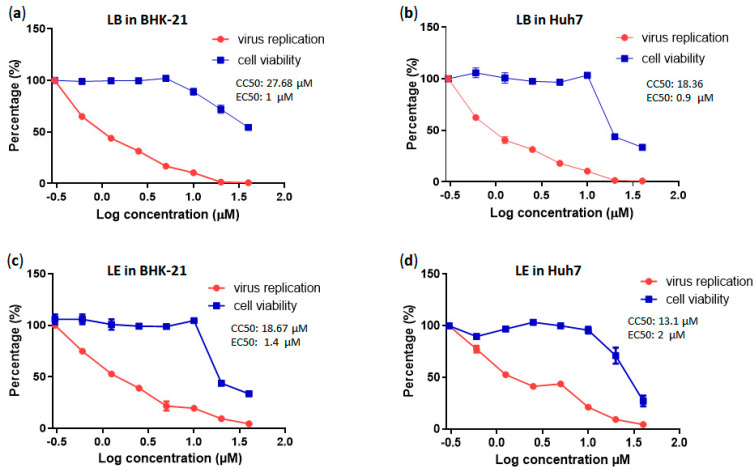
Concentration-response curves for LB and LE. Cells were incubated with CHIKV-CMV-NLuc at an MOI of 0.1 for BHK-21, and an MOI of 2 for Huh7, and treated with liposomes containing berberine or emodin at 2-fold serial dilutions for 16 h. (**a**) Concentration-response curves of liposomes containing berberine in BHK-21cells. (**b**) Concentration-response curves of liposomes containing berberine in Huh7 cells (**c**) Concentration-response curves of liposomes containing emodin in BHK-21 cells. (**d**) Concentration-response curves of liposomes containing emodin in Huh7cells. Black circles represent the luminescence signal, whereas cell viability is represented by black squares. In both cases, the values for mock-treated cells were set to 100%. The data represent the average of three independent experiments. Error bars represent the standard deviation.

A dose-dependent assay demonstrated that LB inhibited CHIKV replication in a dose-dependent manner in both BHK-21 and Huh7 cells. In BHK-21 cells, EC_50_ was ≈1 µM, with a CC_50_ of ≈28 µM, and a selectivity index (SI, defined as the ratio between a compound’s cytotoxic concentration and its effective bioactive concentration) of 28. In Huh7 cells, the EC_50_ was ≈0.9 µM, the CC_50_ was ≈18.4 µM, and the SI was 20.4. Similarly, LE also inhibited CHIKV replication in a dose-dependent fashion, with an EC_50_ of ≈1.4 µM, a CC_50_ of ≈18.7 µM, and an SI of 13.3 in BHK-21 cells and an EC_50_ of ≈2 µM, a CC_50_ of ≈13.1 µM, and an SI of 6.5 in Huh7 cells (Figure 4).

An ideal therapeutic compound should exhibit a relatively high cytotoxic concentration and a very low effective concentration. A compound or drug with an SI ≥ 10 is generally considered to have promising potential for further investigation [71]. The SI values for the LB cells exceeded 20, which is consistent with the findings of previous studies performed using free berberine [11,72]. Notably, to date, there are no published data on dose-dependent studies involving CHIKV-infected BHK-21 or Huh7 cells treated with either encapsulated or free emodin. Our data suggest that, despite the lower SI values, LE may also be considered a potential inhibitor of CHIKV.

### 3.6. LB and LE Lack Virucidal Activity

**Figure 5:**
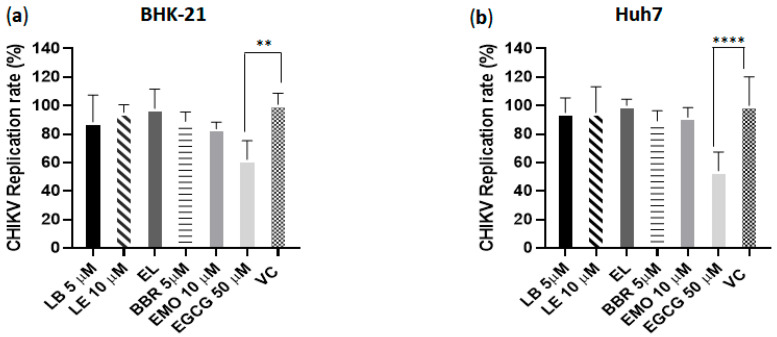
Analysis of virucidal activity. CHIKV-NLuc virions were incubated for 1 h at 37 °C with berberine (BBR), emodin (EMO), epigallocatechin gallate (EGCG), vehicle control (VC, DMSO), LB, LE, and EL, and then used to infect BHK-21 (**a**) or Huh7 (**b**) cells. Cells were harvested, and nanoluciferase activity was measured at 16 hpi. Data represents the average of three independent experiments; error bars indicate the standard deviation. LB: liposome containing berberine; EL: empty liposome; LE: liposome containing emodin; BBR: free berberine; EMO: free emodin; EGCG: epigallocatechin gallate used as a positive control of the assay. VC: vehicle control (DMSO); **: *p* ≤ 0.01; ****: *p* ≤ 0.0001 (one-way ANOVA followed by Tukey’s post-hoc test).

To evaluate the virucidal activities of LB and LE, CHIKV-NLuc was incubated with the compounds for 1 h and then used to infect BHK-21 and Huh7 cells. Treatment with EGCG, used as a positive control, significantly reduced the infectivity of CHIKV-NLuc particles compared with the vehicle control in BHK-21 (*p* ≤ 0.01) and Huh7 cells (*p* ≤ 0.0001). In contrast, free berberine and emodin did not exhibit virucidal activity. Similarly, no reduction in infectivity was observed in samples treated with LB or LE compared with those treated with EL (empty liposomes) (Figure 5). These findings are consistent with previous studies reporting that CHIKV particles were not affected by free berberine at concentrations comparable to those used in our experiments [11,72]. In contrast, emodin in its free form has been shown to exhibit virucidal activity against Zika virus (ZIKV, an *Orthoflavivirus*) when incubated at 40 µM [48]. The lack of virucidal effect against CHIKV may originate from the lower concentrations and/or different properties of CHIKV and ZIKV virions.

### 3.7. Time-of-Addition Assays Using Berberin, Emodin, LB, and LE

Time-of-addition experiments were performed to determine the stage (s) at which the compounds suppressed CHIKV replication. Liposomes containing berberine (5 µM) or emodin (10 µM), as well as the free forms of these compounds, were administered either 2 or 1 h before infection, simultaneously with infection, at 1, 2, 4, 6, and 8 h post-infection (hpi). EGCG (50 µM) was used as a positive control.

**Figure 6:**
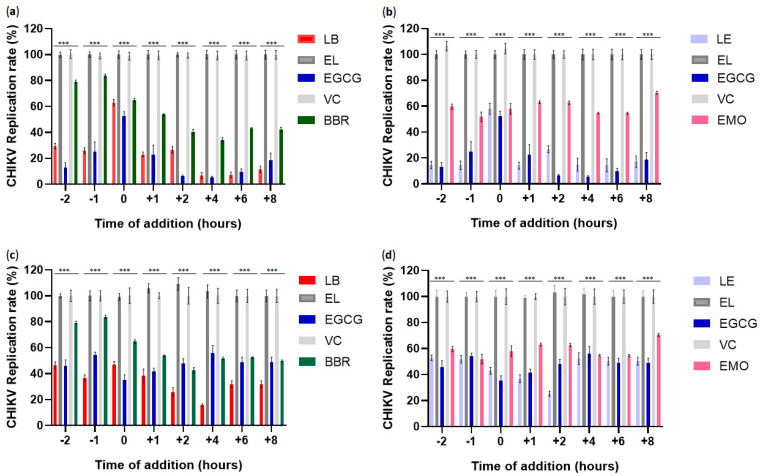
Time-addition assay of compounds in BHK-21 and Huh7 cells. Cells were pre-incubated with the compounds for 2 or 1 h (time points −2 and −1, respectively), treated simultaneously with the virus (time point 0; MOI 0.1 for BHK-21 cells (**a**,**b**) and MOI 2 for Huh7 cells (**c**,**d**)), or treated at 1, 2, 4, 6, or 8 hpi (time points +1, +2, +4, +6, and +8, respectively). Cells were harvested at 16 hpi, and nanoluciferase activity was measured. Values for cells treated with VC were set to 100%. Data represents the average of three independent experiments; error bars indicate standard deviation. LB: liposome containing berberine; EL: empty liposome; LE: liposome containing emodin; BBR: free berberine; EMO: free emodin; EGCG: epigallocatechin gallate used as a positive control of the assay. VC: vehicle control (DMSO); ***: *p* ≤ 0.001 (one-way ANOVA followed by Tukey’s post hoc test).

Liposomal berberine inhibited approximately 70% of CHIKV infection in BHK-21 cells (*p* ≤ 0.001) and 57% in Huh7 cells (*p* ≤ 0.001) at −2 h. A similar inhibitory pattern was observed at -1 h, with reductions of approximately 78% in BHK-21 cells (*p* ≤ 0.001) and 62% in Huh7 cells (*p* ≤ 0.001) (Figure 6a,b). Under the same pretreatment conditions, liposomal emodin inhibited approximately 83% of CHIKV infection in BHK-21 cells (*p* ≤ 0.001) and 50% in Huh7 cells (*p* ≤ 0.001) at −2 h, and 84% (*p* ≤ 0.001) and 50% (*p* ≤ 0.001), respectively, at −1 h (Figure 6c,d). Notably, liposomal berberine was significantly more effective than free berberine in reducing CHIKV infection in both cell lines (*p* ≤ 0.001), whereas liposomal emodin outperformed free emodin only in BHK-21 cells (*p* ≤ 0.001). EGCG, used as a positive control, significantly inhibited CHIKV infection under pretreatment conditions at −2 and −1 h in both cell lines compared to the vehicle control (*p* ≤ 0.001).

CHIKV entry into host cells is mediated by transmembrane glycoproteins E1 and E2, which form trimeric heterodimers that bind to host cell attachment factors and receptors, including the adhesion molecule Mxra8 [73]. Interestingly, Mxra8 expression was downregulated in berberine-treated HS633T cells [74]. A plausible hypothesis for the reduced infection rate observed in berberine-treated cells is that berberine downregulates Mxra8 expression, thereby impairing viral binding. However, this explanation is inconsistent with the experimental timeframe, as a 1 h pre-treatment is likely too short to cause functionally significant changes in Mxra8 levels, suggesting the existence of alternative mechanisms. Emodin’s antiviral activity may parallel that of berberine, as treatment of pig alveolar macrophages with emodin significantly reduced African swine fever virus (ASFV) mRNA levels, suggesting interference with viral binding to host receptors [75]. Furthermore, Batista et al. (2019) reported 42.3% inhibition of ZIKV infection in Vero E6 cells pre-treated with emodin [20].

Simultaneous application of the virus and compounds revealed that liposomes containing berberine or emodin significantly inhibited CHIKV infection compared to empty liposomes in both BHK-21 and Huh7 cells (*p* ≤ 0.001). However, in BHK-21 cells, the liposomal formulations and free forms of the compounds showed similar, relatively low efficacy. In contrast, in Huh7 cells, both LB and LE exhibited significantly higher antiviral activity than free forms of the compounds (*p* ≤ 0.05) (Figure 6c,d). EGCG also inhibited CHIKV infection compared to the vehicle control (*p* ≤ 0.001) in both cell types when the virus was applied simultaneously with EGCG.

One potential mechanism by which berberine and emodin inhibit CHIKV entry is by inhibiting endosomal fusion. Within endosomes, acidic pH triggers fusion between the viral envelope glycoprotein E1 and the endosomal membrane. Thus, an acidic environment is essential for internalization and fusion in CHIKV and other viruses such as ASFV [75,76]. A previous study showed that emodin inhibits ASFV infection by preventing endosomal acidification [75]. Saha et al. (2014) also reported that berberine disrupts the microtubule network in HeLa cells [77]. If the compound exerts a similar effect in Huh7 cells, it may potentially impair CHIKV infectivity by disrupting microtubule dynamics, which is important at early stages of infection [78]. Finally, it is also possible that the relatively modest effect observed in BHK-21 cells is not related to the entry process. Instead, the compound absorbed by the cells may not have been entirely removed by washing and could be affecting subsequent steps of viral infection.

Liposomal formulations of berberine and emodin indeed significantly inhibited CHIKV replication when administered at 1, 2, 4, 6, and 8 hpi compared to empty liposomes (*p* ≤ 0.001). LB exhibited greater inhibition than free berberine during the post-entry stages in BHK-21 (*p* ≤ 0.001 at 1, 4, 6, and 8 hpi and *p* ≤ 0.05 at 2 hpi) cells, as well as at nearly all time points in Huh7 cells (*p* ≤ 0.05 at 2 and 8 hpi; *p* ≤ 0.001 at 4 hpi; *p* ≤ 0.01 at 6 hpi). Similarly, LE demonstrated superior post-entry inhibition compared to free emodin in BHK-21 cells at all times analyzed (*p* ≤ 0.001) and at 1, 2, and 8 hpi in Huh7 cells (*p* ≤ 0.001 at 1 and 2 hpi; *p* ≤ 0.01 at 8 hpi) (Figure 6c,d). EGCG presented significant inhibition in all time points analyzed in the post-entry stages in both cell lines (*p* ≤ 0.001).

Berberine has been shown to inhibit post-entry stages of *Alphavirus* and *Orthoflavivirus* replication by interfering with RNA synthesis and the expression of nonstructural and virion proteins [11,72,74]. CHIKV replication proceeds rapidly, and at an MOI of 5, progeny virions can be detected by approximately 5 hpi and are released around 6 hpi [79]. In our study, the timing of berberine administration suggests that it may disrupt nonstructural protein expression at the stage of infection that corresponds to formation and maturation of the replicase complex and inhibit the synthesis of genomic and subgenomic RNAs [76]. At 6 and 8 hpi, berberine may also interfere with the final steps of the CHIKV replication cycle. Consistently, 3 µM berberine has inhibited CHIKV particle production in BHK-21 cells [72]. However, another study reported that significantly higher concentrations (>50 µM) of free berberine are required to achieve a marked reduction in CHIKV infectivity [80].

Although several studies have reported the antiviral activity of emodin against viruses such as the hepatitis B virus, enterovirus 71, and dengue virus [19,81,82], little is known about its mechanism of action during the post-entry stages of infection. Therefore, further investigation into the mechanisms by which emodin acts during *Alphavirus* infection represents an important avenue for future research.

### 3.8. Antiviral Effects of LB and LE on CHIKV RNA Replication

For *Alphaviruses*, only the nonstructural proteins (nsP1–nsP4) are essential for viral RNA replication [83], allowing the construction of subgenomic replicons in which the structural protein-coding region is replaced with reporter genes and selection markers [84]. Stable cell lines harboring such replicons are increasingly used in the analysis of antivirals targeting the RNA replication stage of the viral life cycle [72,84]. BHK-CHIKV-NCT cells stably express a CHIKV subgenomic replicon that produces *Renilla* luciferase (Rluc), the activity of which is proportional to the replication of the CHIKV NCT replicon [41].

**Figure 7:**
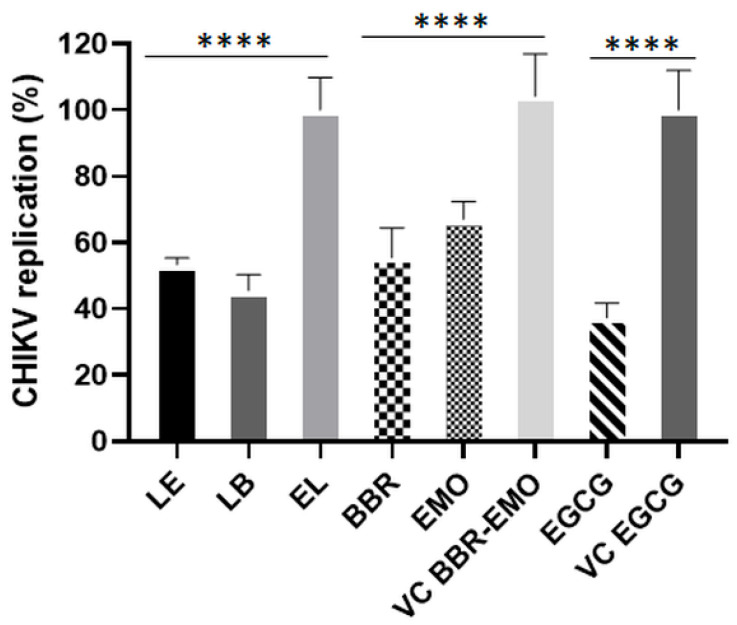
Liposomes containing berberine or emodin inhibited CHIKV RNA replication. BHK-CHIKV NCT cells were seeded 24 h before treatment and treated with liposomes containing berberine or emodin and free berberine and emodin for 48 h. Renilla luciferase activity was measured after this period. Activities observed in the presence of LB and LE were normalized to those in the presence of empty liposomes (EL), whereas the activities of the free compounds were normalized to the vehicle control (VC). Data represent mean values ± standard deviation from three independent experiments performed in quadruplicate. LB: liposome containing berberine; EL: empty liposome; LE: liposome containing emodin; BBR: free berberine; EMO: free emodin; EGCG: epigallocatechin gallate used as a positive control of the assay. VC BBR-EMO: BBR and EMO vehicle control (DMSO); VC EGCG: epigallocatechin gallate vehicle control (DMSO); ****: *p* ≤ 0.0001 (one-way ANOVA followed by Tukey’s post hoc test).

To analyze their effect on CHIKV RNA replication, BHK-CHIKV-NCT cells were treated with compounds for 48 h. Cytotoxicity assessment revealed that these cells tolerated treatment with LB and LE at 5 µM. At this concentration, both berberine- and emodin-containing liposomes significantly inhibited CHIKV RNA replication relative to the empty liposomes, confirming that LB and LE interfere with post-entry stages of the viral infection cycle (*p* ≤ 0.0001). Free berberine and emodin also significantly reduced the expression of nonstructural proteins compared to that in vehicle-treated controls (*p* ≤ 0.0001). However, the inhibition was slightly less pronounced than that observed for LB and LE (*p* ≤ 0.05) (Figure 7). EGCG also significantly inhibited CHIKV RNA replication compared to that of the vehicle control (*p* ≤ 0.0001).

These findings are consistent with the results of the time-of-addition assays (Figure 6) and confirm that the compounds analyzed inhibit CHIKV RNA replication. In contrast, a previous study using free berberine did not report a reduction in luciferase activity in CHIKV replicon cells, suggesting that berberine does not inhibit viral replicase function [85]. The discrepancy between that study and our findings may be attributed to the differences in berberine concentration and treatment duration. Furthermore, our study used berberine-loaded liposomes, which may have enhanced intracellular delivery and increased antiviral efficacy.

### 3.9. Analysis of CHIKV nsP2, nsP3 and Capsid Protein Expression

**Figure 8:**
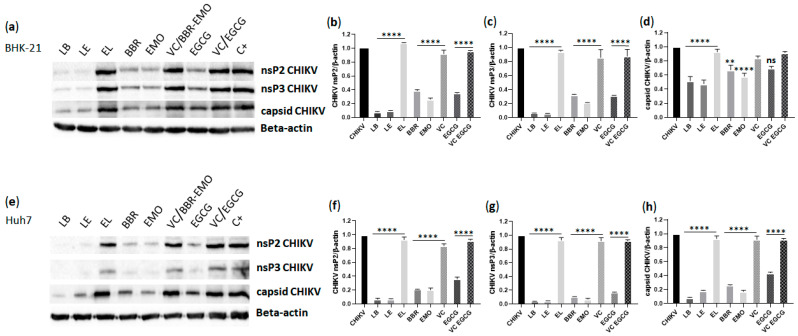
Treatment with LB and LE reduced the expression of CHIKV proteins in BHK-21 and Huh7 cells. Western blot analysis of CHIKV nsP2, nsP3, and capsid proteins in BHK-21 cells (**a**). Quantification of CHIKV nsP2 (**b**), nsP3 (**c**), and capsid protein (**d**) expression in BHK-21 cells. Western blot analysis of CHIKV nsP2, nsP3, and capsid proteins in Huh7 cells (**e**). Quantification of CHIKV nsP2 (**f**), nsP3 (**g**), and capsid protein (**h**) expression in Huh7 cells. β-actin was used as an endogenous control and for normalization in protein quantification. Protein levels in the presence of LB and LE were normalized to those in the presence of empty liposomes (EL), whereas levels in the presence of free compounds were normalized to the vehicle control (VC). Data represent mean values ± standard deviation from three independent experiments performed in quadruplicate. LB: liposomes containing berberine. LE: liposomes containing emodin. EL: empty liposome. BER: free berberine. EMO: emodin. VC/BBR-EMO: Berberine and emodin vehicle control (DMSO). EGCG: (-)-epigallocatechin 3-gallate. VC/EGCG: EGCG vehicle control (DMSO); C+: Positive control (CHIKV-infected cells).**: *p* ≤ 0.01; ****: *p* ≤ 0.0001; ns: non-significant (one-way ANOVA followed by Tukey’s post hoc test).

The impact of the compounds on CHIKV protein expression in BHK-21 and Huh7 cells was analyzed using western blotting. Compared to treatment with empty liposomes, treatment with LB and LE significantly reduced nsP2 and nsP3 expression in both cell lines (*p* ≤ 0.0001) (Figure 8). Treatment with free berberine or emodin also led to a significant decrease in the expression of nsP2 and nsP3 proteins compared to that in vehicle-treated controls (*p* ≤ 0.0001). Notably, LB and LE treatments were more effective than the corresponding free compounds in BHK-21 cells, once LB and LE presented significant decrease in protein expression compared to free compounds for nsP2 protein (LB × BBR and LE × EMO: *p* ≤ 0.0001 in both cell lines) and nsP3 protein (LB × BBR: *p* ≤ 0.001 and LE × EMO: *p* ≤ 0.05 in BHK-21; LB × BBR and LE × EMO: *p* ≤ 0.05 in Huh7) (Figure 8b,c,f,g), likely because of the more efficient cellular uptake of liposomes [86,87].

*Alphavirus* genome replication is initiated and coordinated by the processing of the P1234 polyprotein by the nsP2 protein [88,89], which also exhibits RNA helicase, nucleoside triphosphatase (NTPase), and RNA triphosphatase (RTPase) activities [90]. nsP2 is essential for the formation of the replicase complex (RC) and plays a role in immune evasion, making it a key target for antiviral therapy. Structurally, nsP2 consists of an N-terminal RNA helicase domain (nsP2h) connected via a flexible linker to a C-terminal protease domain (nsP2p) [91]. Our findings showed that all analyzed compounds inhibited nsP2 expression.

One possible mechanism for the reduced levels of mature nsP2 is interference with nsP2-mediated cleavage of the P1234 polyprotein. This also impairs the formation of functional RCs, thereby suppressing RNA replication (Figure 7). However, because ns-protein expression and RNA replication are functionally coupled, the reverse scenario is also plausible—namely, that inhibition of RNA synthesis (through either direct or indirect mechanisms) leads to reduced production of genomic RNA, which also serves as mRNA for ns-protein expression. Between these two possibilities, the latter is more likely, as there is currently no evidence that berberine or emodin act as *Alphavirus* protease inhibitors.

CHIKV nsP3 comprises three functional domains: a macrodomain (MD), which is involved in ADP-ribose binding and hydrolysis; a conserved *Alphavirus* unique domain (AUD), essential for RNA synthesis; and a hypervariable domain (HVD), which mediates extensive interactions with host factors [92]. The AUD domain enables nsP3 to form tubular structures found in mature RCs [92]. In our study, treatment with either LB or LE significantly reduced nsP3 expression in infected cells, mirroring the effects observed with the free compounds. Although these findings suggest that berberine and emodin may interfere with nsP3 functions—such as disrupting AUD-mediated oligomerization required for viral RNA synthesis, inhibiting nsP3-mediated transport of viral RNAs [92], or interfering with interactions with host proteins—an indirect effect, as proposed for nsP2, is more likely.

CHIKV capsid protein expression was also significantly inhibited by both LB and LE compared to EL (*p* ≤ 0.0001), as well as by the free compounds compared to vehicle control (BBR × VC: *p* ≤ 0.01 and EMO × VC: *p* ≤ 0.0001) (Figure 8a,d,e,h). Once again, LB treatment was more effective than free berberine in both cell types (*p* ≤ 0.01 in BHK-21 and *p* ≤ 0.0001in Huh7), whereas LE did not show enhanced efficacy compared to free emodin in BHK-21 and Huh7 cells. EGCG treatment significantly inhibited capsid protein expression only in Huh7 cells (*p* ≤ 0.0001).

The *Alphavirus* capsid protein is multifunctional, containing a disordered N-terminal domain involved in RNA encapsidation and nucleocapsid core assembly [93,94], and a C-terminal serine protease domain essential for structural polyprotein processing [94]. Studies have shown that the capsid protein binds viral RNA at specific sites, potentially regulating the translation of viral proteins [95]. Targeting any of these functions could impact viral replication. However, since the compounds also inhibited replication of the CHIKV NCT replicon (Figure 7), which lacks the structural region, it is unlikely that the capsid protein is a primary target. Instead, the observed reduction in capsid expression was a downstream consequence of reduced genomic and subgenomic RNA synthesis.

### 3.10. Liposome Containing Berberine or Emodin Do Not Intercalate with Double-Stranded RNA (dsRNA)

**Figure 9:**
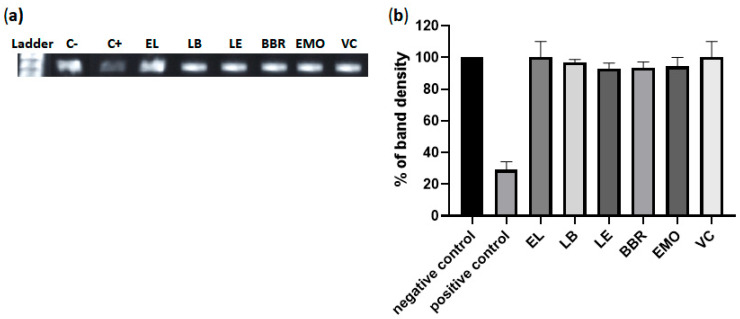
Liposomes containing berberine or emodin do not interact with dsRNA. (**a**) A 1% agarose gel showing representative results; dsRNA was visualized using ethidium bromide staining. (**b**) Quantification of band density, expressed as the percentage of treated RNA relative to the negative control (cells without any treatment). Doxorubicin (100 µM) was used as a positive control (C+). Data represent mean values ± standard deviation from three independent experiments. LB: liposomes containing berberine; LE: liposomes containing emodin; EL: empty liposome. BER: free berberine; EMO: free emodin; VC: vehicle control (DMSO); positive control: doxorubicin; negative control: RNA without any treatment.

To further investigate the mechanism underlying the anti-CHIKV activity of liposomes containing berberine or emodin, we assessed their ability to intercalate into dsRNA. A 350 bp dsRNA fragment corresponding to a truncated 3′ untranslated region of CHIKV was generated via in vitro transcription and strand annealing. This dsRNA was incubated with liposomes containing 5 µM berberine or 10 µM emodin, their respective free forms at equivalent concentrations, or doxorubicin, a known dsRNA intercalator. The gel retardation assay revealed that neither liposome-encapsulated berberine nor emodin, nor their free forms, exhibited intercalation into dsRNA, in contrast to the positive control, doxorubicin (Figure 9). Therefore, the inhibition of viral replication by these liposomes cannot be attributed to interactions with dsRNA, which serves as a replication intermediate.

## 4. Conclusions

Liposomes containing berberine or emodin inhibit chikungunya virus (CHIKV) infection in cell culture and are more effective than the free forms of these compounds. The results of this study demonstrate that liposomal encapsulation of natural compounds can enhance their antiviral activity against CHIKV. Encapsulation in liposomal systems not only improves the bioavailability of these compounds but also enables a more targeted and efficient antiviral response. The compounds act at multiple stages of CHIKV infection, including post-entry phases, and are also effective in prophylactic treatment. These findings highlight the promising role of nanotechnology in developing innovative antiviral therapies, particularly for arboviruses for which no specific treatments are currently available. Further studies are warranted to elucidate the underlying molecular mechanisms and to validate the in vivo efficacy of these liposomal formulations.

## Figures and Tables

**Table 1:** Average of hydrodynamic size and zeta potential for empty liposome (EL), liposome containing emodin (LE), and liposome containing berberine (LB).

Liposome	Average Size (nm)	Average Zeta Potential (mV)
EL	135 ± 7	−14 ± 2
LE	206 ± 7	−19 ± 2
LB	155 ± 5	−15 ± 2

## Data Availability

The original data presented in the study are openly available at https://repositorio.unesp.br/home (accessed on 20 August 2025).

## Supplementary Materials

The following supporting information can be downloaded at: https://www.mdpi.com/article/10.3390/pharmaceutics17091229/s1. Figure S1. Cytotoxicity analysis of liposomes containing berberine, liposomes containing emodin and empty liposomes in BHK-21 and HuH-7.0 cells.
