# Supplementary material for: Antiviral Activity of Liposomes Containing Natural Compounds Against CHIKV
Source: Pharmaceutics. 2025 Sep 22;17(9):1229. doi: 10.3390/pharmaceutics17091229 (PMC12473542; doi:10.3390/pharmaceutics17091229)
Supplement: Supplementary file 1 [file pharmaceutics-17-01229-s001.zip › pharmaceutics-3858312-SI.pdf]

Antiviral activity of liposomes containing natural compounds against CHIKV

Supplementary figures

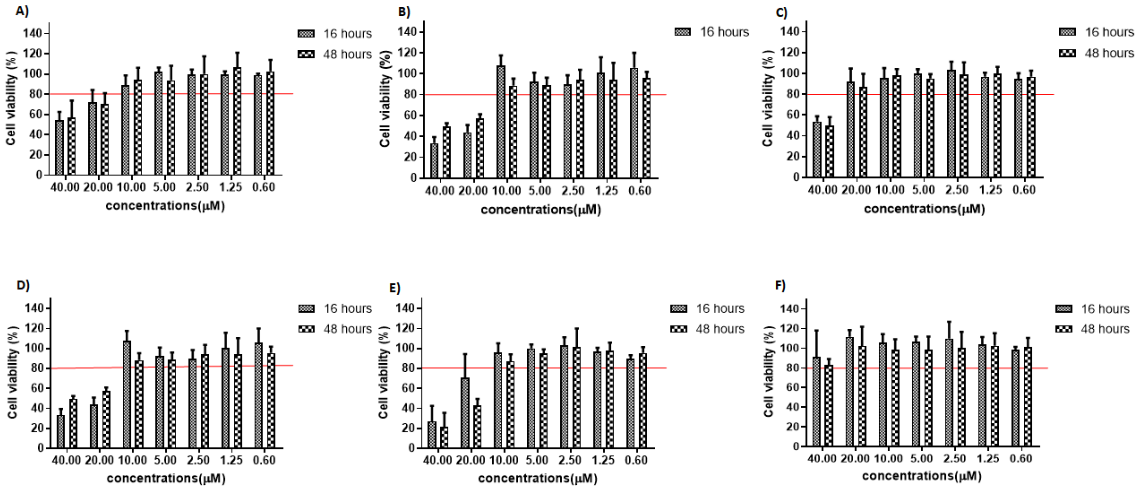

Supplementary Figure S1. Cytotoxicity analysis of liposomes containing berberine, liposomes containing emodin and empty liposomes in BHK-21 and Huh7 cells. (**A**) Cytotoxicity analysis of liposomes containing berberine at concentrations of 0.60 to 40 μM incubated in BHK-21 cells for 16 and 48 hours. (**B**) Cytotoxicity analysis of liposomes containing emodin at concentrations of 0.60 to 40 μM incubated in BHK-21 cells for 16 and 48 hours. (**C**) Cytotoxicity analysis of empty liposomes at concentrations of 0.60 to 40 μM incubated in BHK-21 cells for 16 and 48 hours. (**D**) Cytotoxicity analysis of liposomes containing berberine at concentrations of 0.60 to 40 μM incubated in HUH-7 cells for 16 and 48 hours. (**E**) Cytotoxicity analysis of liposomes containing emodin at concentrations of 0.60 to 40 μM incubated in Hhu7 cells for 16 and 48 hours. (**F**) Cytotoxicity analysis of empty liposomes at concentrations of 0.60 to 40 μM incubated in HUH-7.0 cells for 16 and 48 hours.
